# Supplementary material for: Exploratory Factor Analysis (EFA) of the Short Functional Geriatric Evaluation (SFGE) to Assess the Multidimensionality of Frailty in Community-Dwelling Older Adults
Source: Int J Environ Res Public Health. 2023 Feb 25;20(5):4129. doi: 10.3390/ijerph20054129 (PMC10001926; doi:10.3390/ijerph20054129)
Supplement: Supplementary file 1 [file ijerph-20-04129-s001.zip › ijerph-2033249-supplementary.pdf]

Correlation Matrix

		q1	q2	q3	q4	q5	q6	q7	q8	q9	q10	q11	q12
**Correlation**	q1	1.000	0.058	-0.152	-0.051	0.050	-0.080	-0.042	0.108	0.255	0.208	0.113	0.128
	q2	0.058	1.000	-0.008	0.013	0.160	-0.013	0.179	0.056	0.126	0.143	0.069	0.066
	q3	-0.152	-0.008	1.000	0.176	0.044	0.139	0.016	-0.142	-0.303	-0.217	-0.200	-0.158
	q4	-0.051	0.013	0.176	1.000	0.041	0.056	0.182	-0.019	-0.122	-0.081	-0.073	-0.055
	q5	0.050	0.1.0	0.044	0.041	1.000	0.085	0.072	0.065	0.077	0.105	-0.004	0.056
	q6	-0.080	-0.013	0.139	0.056	0.085	1.000	-0.028	-0.191	-0.288	-0.218	-0.239	-0.178
	q7	-0.042	0.179	0.016	0.182	0.072	-0.028	1.000	0.084	0.074	0.060	0.021	0.037
	q8	0.108	0.056	-0.142	-0.019	0.065	-0.191	0.084	1.000	0.372	0.316	0.278	0.430
	q9	0.255	0.126	-0.303	-0.122	0.077	-0.288	0.074	0.372	1.000	0.575	0.388	0.349
	q10	0.208	0.143	-0.217	-0.081	0.105	-0.218	0.060	0.316	0.575	1.000	0.437	0.321
	q11	0.113	0.069	-0.200	-0.073	-0.004	-0.239	0.021	0.278	0.388	0.437	1.000	0.294
	q12	0.128	0.066	-0.158	-0.055	0.056	-0.178	0.037	0.430	0.349	0.321	0.294	1.000
**Sig. (1-tailed)**	q1		0.000	0.000	0.000	0.000	0.000	0.000	0.000	0.000	0.000	0.000	0.000
	q2	0.000		0.226	0.115	0.000	0.112	0.000	0.000	0.000	0.000	0.000	0.000
	q3	0.000	0.226		0.000	0.000	0.000	0.070	0.000	0.000	0.000	0.000	0.000
	q4	0.000	0.115	0.000		0.000	0.000	0.000	0.038	0.000	0.000	0.000	0.000
	q5	0.000	0.000	0.000	0.000		0.000	0.000	0.000	0.000	0.000	0.343	0.000
	q6	0.000	0.112	0.000	0.000	0.000		0.004	0.000	0.000	0.000	0.000	0.000
	q7	0.000	0.000	0.070	0.000	0.000	0.004		0.000	0.000	0.000	0.024	0.000
	q8	0.000	0.000	0.000	0.038	0.000	0.000	0.000		0.000	0.000	0.000	0.000
	q9	0.000	0.000	0.000	0.000	0.000	0.000	0.000	0.000		0.000	0.000	0.000
	q10	0.000	0.000	0.000	0.000	0.000	0.000	0.000	0.000	0.000		0.000	0.000

q11	0.000	0.000	0.000	0.000	0.343	0.000	0.024	0.000	0.000	0.000		0.000
q12	0.000	0.000	0.000	0.000	0.000	0.000	0.000	0.000	0.000	0.000	0.000	

Supplementary material

Supplementary Table S1. Correlation Matrix.

Supplementary material - Methodology

There is at present an open discussion about the use of PCA with varimax rotation for EFA ("despite several authors still remain using it…"). The technique presents with several limitations (that we have highlighted among the limitations of our study).

However, we quote here the argument of Brian P. O'Connor, who developed the SPSS syntax for the parallel analysis: "Principal components eigenvalues are often used to determine the number of common factors. This is the default in most statistical software packages, and it is the primary practice in the literature. It is also the method used by many factor analysis experts, including Cattell, who often examined principal components eigenvalues in his scree plots to determine the number of common factors. But others believe this common practice is wrong. […] The issue remains neglected and unsettled.

For example, the method *"Exploratory factor analysis using principal component analysis with varimax rotation"* in relation to questionnaires was used by some researchers in different papers published recently:

1) Bampa G, Kouroglou D, Metallidou P, Tsolaki M, Kougioumtzis G, Papantoniou G, Sofologi M, Moraitou D. Metacognitive Scales: Assessing Metacognitive Knowledge in Older Adults Using Everyday Life Scenarios. Diagnostics (Basel). 2022 Oct 5;12(10):2410. doi: 10.3390/diagnostics12102410. PMID: 36292099; PMCID: PMC9600082.

2) Sollid MIV, Slaaen M, Danielsen S, Kirkevold Ø. Psychometric properties of the person-centred coordinated care experience questionnaire (P3CEQ) in a Norwegian radiotherapy setting. Int J Qual Health Care. 2022 Sep 15;34(3):mzac067. doi: 10.1093/intqhc/mzac067. PMID: 36004618; PMCID: PMC9475430.

3) Nikolic A, Bukurov B, Kocic I, Soldatovic I, Mihajlovic S, Nesic D, Vukovic M, Ladjevic N, Grujicic SS. The Validity and Reliability of the Serbian Version of the Smartphone Addiction Scale-Short Version. Int J Environ Res Public Health. 2022 Jan 22;19(3):1245. doi: 10.3390/ijerph19031245. PMID: 35162268; PMCID: PMC8835088.

4) Wong LP, Alias H, Danaee M, Lee HY, Tan KM, Tok PSK, Muslimin M, AbuBakar S, Lin Y, Hu Z. Assessment of Impact of Containment During the COVID-19 Epidemic and Coping Behaviours Using Newly Developed Assessment Tools. Front Public Health. 2021 Dec 22;9:787672. doi: 10.3389/fpubh.2021.787672. PMID: 35004587; PMCID: PMC8728738.

5) Ishizaki, T., Masui, Y., Nakagawa, T., Yoshida, Y., Ishioka, Y. L., Hori, N., Inagaki, H., Ito, K., Ogawa, M., Kabayama, M., Kamide, K., Ikebe, K., Arai, Y., & Gondo, Y. (2022). Construct Validity of a New Health Assessment Questionnaire for the National Screening Program of Older Adults in Japan: The SONIC Study. *International Journal of Environmental Research and Public Health*, *19*(16), 10330. https://doi.org/10.3390/ijerph191610330

Not all the results of the procedures have been presented in the study for the unique reason that the manuscript had to respect word and table limitations.

results of parallel analysis,
Parallel analysis had been computed. We did not present it because it confirmed the validity of the Principal Components Eigenvalues:

Varianza totale spiegata

Componente	Autovalori iniziali			Caricamenti somme dei quadrati di estrazione			Caricamenti somme dei quadrati di rotazione		
	[illegible]	% di varianza	% cumulativa	Totale	% di varianza	% cumulativa	Totale	% di varianza	% cumulativa
1	2,944	24,537	24,537	2,944	24,537	24,537	2,770	23,081	23,081
2	1,390	11,584	36,121	1,390	11,584	36,121	1,354	11,287	34,369
3	1,106	9,213	45,334	1,106	9,213	45,334	1,316	10,965	45,334

These are the PC Eigenvalues

```
PARALLEL ANALYSIS:

Principal Axis / Common Factor Analysis

Specifications for this Run:
Ncases      8800
Nvars         12
Ndatsets     100
Percent       95

Random Data Eigenval[illegible]
        Root           Means      Prcntyle
     1.000000        .060175       .075547
     2.000000        .047081       .056299
     3.000000        .034735       .043980
```

These are the Random Data Eigenvalues, calculated by means of the Parallel Analysis.

As shown, Random Data Eigenvalues are always SMALLER than the Initial Eigenvalues, confirming the validity of the new components.
But also, this interpretation is a matter of discussion (see: Buja, A., & Eyuboglu, N., 1992, Remarks on parallel analysis. Multivariate Behavioral Research, 27, 509-540.): "Parallel analyses of adjusted correlation matrices eg, with SMCs on the diagonal, tend to indicate more factors than warranted. The eigenvalues for trivial, negligible factors in the real data commonly surpass corresponding random data eigenvalues for the same roots.

comparison between pattern matrix and rotated matrix,

Matrice dei componenti[a]

	Componente		
	1	2	3
q1_N	,358	-,084	,426
q2_N	,199	,532	,346
q3_N	-,440	,329	-,173
q4_N	-,177	,532	-,417
q5_N	,104	,501	,516
q6_N	-,445	,168	,378
q7_N	,099	,634	-,264
q8_N	,610	,122	-,244
q9_N	,788	,000	,069
q10_N	,745	,072	,084
q11_N	,640	-,062	-,148
q12_N	,613	,057	-,171

Metodo di estrazione: Analisi dei componenti principali.

a. 3 componenti estratti.

Matrice dei componenti ruotati[a]

	Componente		
	1	2	3
q1_N	,208	-,417	,316
q2_N	,098	,122	,646
q3_N	-,356	,452	,012
q4_N	-,025	,698	,033
q5_N	-,045	,012	,726
q6_N	-,534	-,017	,288
q7_N	,193	,611	,266
q8_N	,658	,106	,038
q9_N	,728	-,225	,212
q10_N	,685	-,172	,262
q11_N	,652	-,097	-,014
q12_N	,637	,011	,045

Metodo di estrazione: Analisi dei componenti principali.
Metodo di rotazione: Varimax con normalizzazione Kaiser.[a]

a. Convergenza per la rotazione eseguita in 5 iterazioni.

Here, comparison is presented. The Component Matrix did not show the ideal situation in which one variable measures only one component. As this was not the case, we proceeded with performing a rotation. In the Rotated Component Matrix one variable is linked to one component. We considered here absolute values>0.45 **with the only exception of age;**

commonalities,

Comunalità

	Iniziale	Estrazione
q1_N	1,000	,317
q2_N	1,000	,442
q3_N	1,000	,331
q4_N	1,000	,488
q5_N	1,000	,529
q6_N	1,000	,369
q7_N	1,000	,481
q8_N	1,000	,446
q9_N	1,000	,626
q10_N	1,000	,568
q11_N	1,000	,435
q12_N	1,000	,408

Metodo di estrazione: Analisi dei componenti principali.

Communalities have been computed. No R_2<0.3, therefore no variable was excluded from the analysis.

quality of factor scores,

Quality of Factor Scores has not been directly computed. This is because there is still an open discussion how to actually determine it in the best way. As a proxy, we used the KMO test (which was about 0.8) and the Bartlett test of Sphericity ($p<0.001$).
